# Titanium Dioxide Nanotube-Based Oxygen Indicator for Modified Atmosphere Packaging: Efficiency and Accuracy

**DOI:** 10.3390/ma11122410

**Published:** 2018-11-29

**Authors:** Junwei Wen, Shuting Huang, Yu Sun, Zhengjie Chen, Yixiang Wang, Houbin Li, Xinghai Liu

**Affiliations:** 1 School of Printing and Packaging, Wuhan University, Wuhan 430079, China; 2017202170020@whu.edu.cn (J.W.); 2017301750008@whu.edu.cn (Y.S.); 2017301750025@whu.edu.cn (Z.C.); lhb@whu.edu.cn (H.L.); 2 Faculty of Agricultural and Environmental Sciences, McGill University, Montreal, QC H39 3V9, Canada; shuting.huang@mail.mcgill.ca (S.H.); yixiang.wang@mcgill.ca (Y.W.)

**Keywords:** titanium dioxide, oxygen indicator, photocatalytic activity, screen-printing method, modified atmosphere packaging

## Abstract

Colorimetric oxygen indicators can be applied for non-destructive testing in packaging; especially in modified atmosphere packaging (MAP). In this paper; titanium dioxide (TiO_2_) nanotube; which is used as a semiconductor photocatalyst in oxygen indicators; was synthesized via a microwave-assisted hydrothermal method. X-Ray Diffraction (XRD) was used to analyze its crystal form and Scanning Electron Microscope (SEM).to characterize its morphology. Its properties were studied using Brunauer-Emmett-Teller (BET), Diffuse Reflection Spectrum (DRS), and Bluebottle experiments. The results showed that the synthesized TiO_2_ nanotube was a mixture of rutile and anatase; with a specific surface area of 190.35 m^2^/g; and a wide band gap of 3.34 eV. Given the satisfactory performance; the TiO_2_-based oxygen indicator was prepared and combined with glycerol; methylene blue; and hydroxyethyl cellulose (HEC). The oxygen indicator demonstrated excellent photocatalytic performance and effectively avoided excitation by visible light. We studied the rheological properties; thixotropic properties; and wettability of the indicator. The results demonstrated the printability of the indicator solution; which was then printed in the polyethylene terephthalate (PET) film by screen printing and applied to MAP. The application results showed that the prepared oxygen indicator was able to provide visual support to judge whether the packaging was intact and the food was safe.

## 1. Introduction

Oxygen indicators are leak indicators that monitor the integrity of modified atmosphere packaging (MAP). Currently, there are two types of oxygen indicator mechanisms: electrochemistry oxygen indicators and optical oxygen indicators. Electrochemistry oxygen indicators have been widely applied to detect the integrity of MAP because they precisely measure the oxygen level. However, electrochemistry indicators are expensive and require professional operation [1,2,3,4]. Also, the electrochemistry testing damages the MAP, leading to non-reusable packaging. Given these problems, researchers have been working on a substitute for electrochemistry indicators. The optical oxygen indicator has potential by providing visible information of present oxygen in MAP, representing a novel approach for detecting packaging integrity [5,6,7].

Generally, optical oxygen indicators are based on the transition of metal oxides combined with some connection material and dye. The indicator is sealed inside the MAP and detect MAP integrity using color changes. The optical oxygen indicator is a nondestructive testing technique that reduces packaging waste. MAP integrity can be detected in real-time without further operation or devices. Consumers can make a judgment through the color change of the indicator. Furthermore, this method is eco-friendly and inexpensive [8,9,10].

In this work, we prepared an optical oxygen indicator based on the transition of a metal oxide, namely titanium dioxide (TiO_2_). It is an ultraviolet (UV) photochromic oxygen indicator incorporating methylene blue (MB), TiO_2_, and glycerin, first introduced in 2002 [11]. Its mechanism is as follows: TiO_2_ produces electrons with high reducibility and positive charge holes after UV irradiation. These electrons are able to reduce MB (blue) into leuco methylene blue (LMB, colorless) and the positive holes are combined with glycerin [9,12]. TiO_2_-based oxygen indicators maintain their color in the absence of oxygen. LMB is oxidized to MB (blue) by oxygen when the packaging is damaged. Thus, TiO_2_-based oxygen indicators can used for visible and nondestructive integrity detection in MAP through color change (from colorless to blue) [13].

However, regular TiO_2_ particles, whose photo-generated electrons and holes can easily recombine, do not appropriately photocatalyze to reduce MB. This limits its efficiency as an oxygen indicator [14,15,16]. TiO_2_ particles have a band gap around 3.2 eV, which can be activated by natural light [17]. These aspects restrict its potential for use as an oxygen indicator in MAP integrity detection by reducing its efficiency and accuracy.

Hence, we attempted to improve the properties of TiO_2_ nanotubes using the microwave-assisted hydrothermal method. Hydroxyethyl cellulose (HEC) was added to the TiO_2_ nanotube/MB/glycerin system, promoting the indicator’s printability. The synthesized TiO_2_ nanotubes had a larger surface area, which improved the photocatalytic performance [18,19]. Additionally, the wider band gap of the product prevents activation by natural light. Thus, the efficiency and accuracy of the oxygen indicator have been improved using TiO_2_ nanotubes as semiconductor photocatalyst.

## 2. Material and Methods

### 2.1. Materials

Titanium sulfate, anhydrous ether, anhydrous ethanol, glycerol, and methylene blue (MB) were purchased from China Pharmaceutical Group Pharmaceutical Co. Ltd. (Shanghai, China) and hydroxyethyl cellulose (HEC) was purchased from Shanghai Aladdin Biochemical Technology Co., Ltd. (Shanghai, China). The polyethylene terephthalate (PET) film was supplied by Hubei Yunhe Salt Industry Packing Co., Ltd. (Wuhan, China). All chemicals were analytical grade and used as received without further purification.

### 2.2. Experimental Section

#### 2.2.1. Synthesis of TiO_2_ Nanotubes

**Figure 1:**
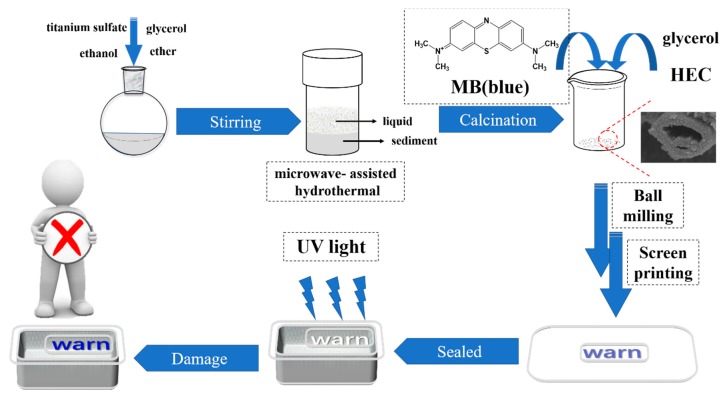
Scheme for TiO_2_ nanotubes processing and application.

The procedure for synthesizing TiO_2_ nanotubes and applying to the oxygen indicator is shown in Figure 1. Titanium sulfate, glycerol, anhydrous ethanol and anhydrous ether were prepared in a molar ratio of 1:16:40:11. Firstly, titanium sulfate was dissolved in anhydrous ethanol, followed by a 30 min ultrasonic dispersion. Then, glycerin and anhydrous ether were added under constant magnetic stirring. After 30 min, the solution was treated with ultrasound (KQ5200E, Kunshan ultrasonic instruments Co., Ltd., Kunshan, China) for another 30 min. The mixed solution was transferred to a Teflon kettle (Sineo Microwave Chemistry Technology Co., Ltd., Shanghai, China) and reacted in a Microwave Digestion System (MDS-6, Sineo Microwave Chemistry Technology Co., Ltd., Shanghai, China). The synthesized products were washed with anhydrous ethanol four times until the impurities were removed, followed by vacuum drying (60 °C, DZF-6021, Yiheng Instrument Co., Ltd., Shanghai, China) overnight. Then, the samples were calcinated for 3 h at 550 °C. The TiO_2_ nanotube samples were obtained through the above process. Table 1 displays four samples with different reaction conditions, labeled T1, T2, T3, and T4.

#### 2.2.2. Blue Bottle Light Experiment

The bluebottle experiment was as follows [20], with moderate modifications. Certain proportions of TiO_2_ and glycerin were added to the MB solution (2%), followed by ultrasound treatment for 30 s. The homogeneous solution was obtained. Then, the absorption spectra of the solution, before and after the UV irradiation with a light intensity of 0.5 mW/cm^2^ (ZF-20D, Shanghai Jinqi Instrument Equipment Co. Ltd., Shanghai, China), were measured using ultraviolet and visible spectrophotometers between 450 nm and 750 nm (UV-3600, Shimadzu Corporation, Kyoto, Japan).

#### 2.2.3. Preparation of Oxygen Indicator

HEC and self-made TiO_2_ powder were dissolved in water with constant stirring until the solution was homogeneous. Then, MB solution (2%) and glycerin were added, followed by a ball mill process. The oxygen indicator solution was obtained, which was printed onto the PET film using screen printer (SYP5, United Engineering Industrial Co. Ltd., Shenzhen, China). After air drying, the PET film was sealed inside the packaging with N_2_ using modified atmosphere packaging machine (DG-300QD, Wuhan Ding Gong Technology Co. Ltd., Wuhan, China).

### 2.3. Characterization

The crystal form of TiO_2_ was measured by X-ray diffraction (XRD, D8, Bruker, Billerica, MA, USA) in the range of 8 to 80° and scanning speed of 6°/s. A scanning electron microscope (SEM, SIGMA, Carl Zeiss Microscopy, Oberkochen, Germany) was used to analyze the samples’ morphology. Metal spraying pretreatment was required. The specific surface area of these samples was examined with a gas sorption analyzer (Autosorb Iq, Quantachrome Instruments, Boynton Beach, FL, USA), with porosity ranging from 3.5 A to 5000 A. The solid samples were dried and ground into uniform powder before tests. Diffuse reflection spectra of samples were measured using UV-visible-near infrared spectrophotometer. Then, the band gap was calculated using the Tauc plot method.

Thixotropy, viscosity, and rheological measurements (rotating rheometer, Kinexus Pro+, Malvern, UK) were used to estimate the printability of oxygen indicator in the PET film. Specifically, thixotropy was carried out in a three-stage method. The shear rate in each section was 0.1 s^−1^, 1000 s^−1^, and 0.1 s^−1^ and the reaction time was 30 s, 30 s, and 10 min, respectively. The viscosity analysis used the single shear rate method with a shear rate of 0.01 s^−1^. The rheological properties were characterized by the changes in viscosity with the shear rate. The initial and terminal shear rates were 0.1 s^−1^ and 1000 s^−1^, respectively. The contact angle of oxygen indicator solution on the PET film was examined using an optical dynamic and static contact angle instrument (SL200, Kino industrial Co., Ltd., Boston, MA, USA).

## 3. Results and Discussion

### 3.1. Characterization of TiO_2_ Nanotube

**Figure 2:**
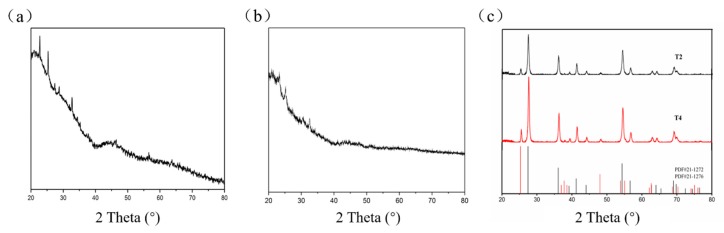
XRD patterns of (**a**) sample T1, (**b**) sample T3, and (**c**) sample T2 and T4.

In order to determine the type of samples, we analyzed their XRD patterns first. The patterns of sample T1 and T3 (without calcination) showed no peaks relating to any crystalline phases, indicating that samples T1 and T3 were amorphous (Figure 2a,b). The calcined patterns of samples T2 and T4 (Figure 2c) exhibited peaks at 27°, 42°, 54°, and 56°, corresponding to the crystal structure of rutile TiO_2_ (PDF 21–1276). The other peaks at 25°, 37°, 55° and 62° corresponded to the crystal structure of anatase TiO_2_ (PDF 21–1272). The results suggest the synthesized products were composed of a mixture of rutile and anatase TiO_2_. Sample T4 (reaction time three hours) exhibited the higher crystallinity with stronger intensity compared to sample T2 (reaction time two hours), suggesting that the longer reaction time and calcination contributed to the formation of crystallization.

Then, we measured the photocatalytic performance of amorphous samples T1 and T3 using the blue bottle light experiment. There were no color changes under the irradiation of UV light. This means samples T1 and T3 were incapable of reducing MB so would not be suitable oxygen indicator. Thus, the attention was paid on the performance study of samples T2 and T4.

**Figure 3:**
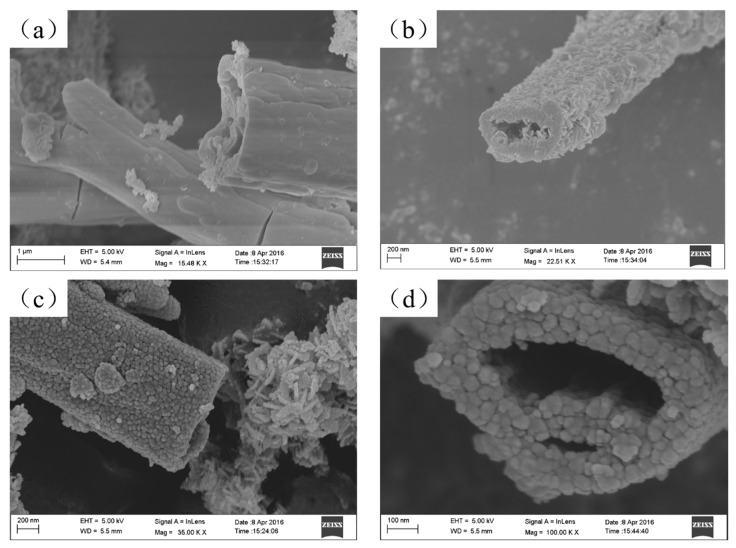
SEM images of (**a**,**b**) sample T2 and (**c**,**d**) sample T4.

SEM images depicted the tube structure of the synthesized samples T2 and T4 (Figure 3a,c, respectively), with uniform shapes and flat surface. Figure 3b (magnification around 22,510×) and d (magnification of 100,000×) revealed that the TiO_2_ nanotubes formed a hollow structure with open ends, additionally formed by piled nanoparticles. The formation of hollow nanotube structure can be attributed to the reaction of SO_4_^2−^ and mixed ether solution under specific conditions. The formed substances promoted the nanoparticles to form a special structure.

**Figure 4:**
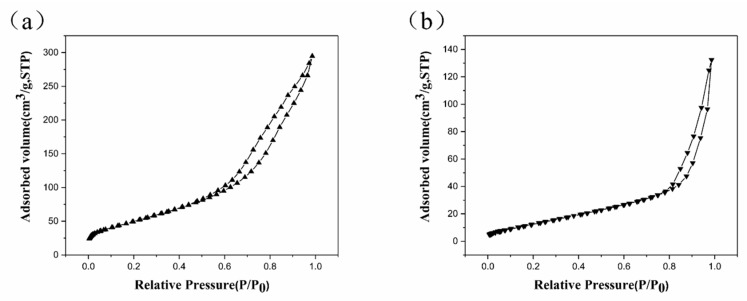
N_2_ adsorption isothermal diagram of (**a**) sample T2 and (**b**) sample T4.

Figure 4 presents the nitrogen adsorption-desorption isotherms of samples T2 and T4. The curves exhibited convexity under low pressure and capillary condensation under high pressure, in agreement with the type IV isotherm. This suggests that both samples were mesoporous material in terms of International union of pure and applied chemistry (IUPAC) classification [21]. The lagging loop could be ascribed to the irreversible adsorption under the equivalent pressure. Specifically, the condensation in the absorption layer of the pore wall occurred simultaneously with the multimolecular layer adsorption. However, the capillary condensation was the only cause of the desorption. The BET-specific surface area of sample T2 was calculated as 55.00 m^2^/g, whereas that of sample T4 was 190.35 m^2^/g, 71% larger than sample T2. This could have resulted from a better degree of crystallization and the particles-built tubular structure. The increase in surface area would benefit from the adsorption of dye, which could increase the photocatalytic activity.

**Figure 5:**
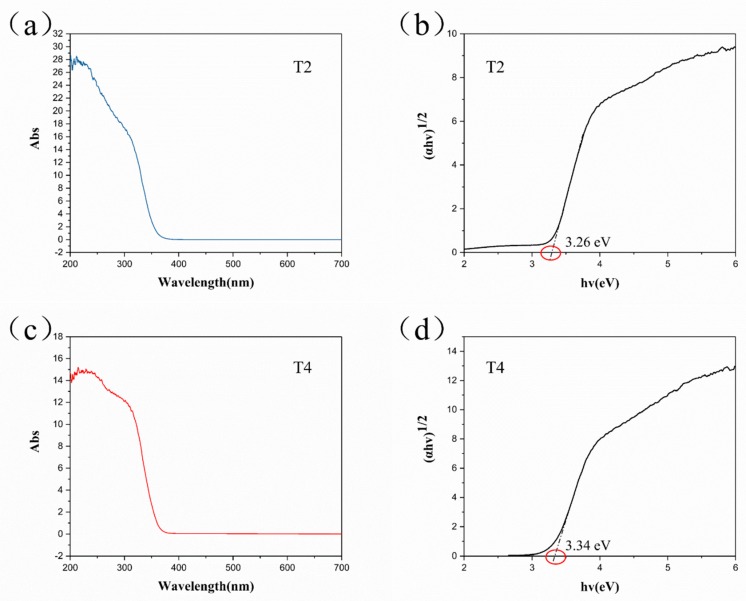
(**a**) UV-vis diffuse reflection and (**b**) calculated energy gap using the Kubelka-Munk equation and Tauc plot method of the as-synthesized samples T2 and T4.

Figure 5 shows the DRS curves of T2 and T4, presenting a blue shift of reflectance. Conversion between reflectance and absorbance was carried out using the Kubelka-Munk equation [22]. The band gap of the samples was calculated using the Tauc plot method as 3.26 eV and 3.34 eV for T2 and T4 (Figure 5b,d), respectively. Samples T2 and T4 both displayed an increase in band gap compared with TiO_2_ particle (TP) (~3.20 eV), which might be attributable to the change in morphology. High excitation energy could avoid being affected by natural light as an oxygen indicator. Specifically, TiO_2_ with a band gap of 3.20 eV can be excited at a wavelength of 380 nm. This means visible light would be sufficient to activate TiO_2_ to reduce MB, producing an unsuitable and inaccurate indicator.

The results of XRD, SEM, BET, and DRS confirmed that TiO_2_ nanotube was successfully synthesized, and sample T4 demonstrated a hollow morphology, larger specific surface area, and a wide band gap. The photocatalytic performance of samples T2 and T4 were measured in the blue bottles experiment.

### 3.2. Characterization of Photocatalytic Properties

**Figure 6:**
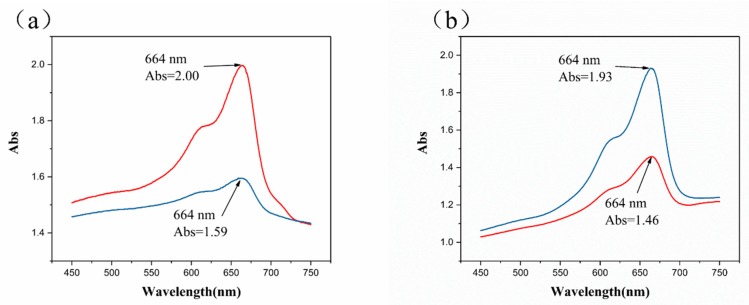
UV absorption spectra of blue bottle solution (before and after UV irradiation for 30 s) for (**a**) sample T2 and (**b**) sample T4.

The photocatalytic performance of samples T2 and T4 during the activation stage were investigated by the reducing ability of MB in the blue bottle experiment. The two solutions were irradiated under the same UV intensity (0.5 mW/cm^2^) for 30 s, and the spectra of the absorbance of MB were recorded before and after irradiation. As shown in Figure 6, UV-vis analysis indicated that MB possessed two specific peaks at 610 nm and 664 nm. After 30 s UV irradiation, the two peaks almost disappeared because MB was reduced to its colorless form (LMB) by photo-generated electrons. The absorbance peak at 664 nm of sample T2 decreased from 2.00 to 1.59 (Figure 6a) after UV irradiation, approaching a drop ratio of 20.5%. The absorbance (664 nm) of sample T4 decreased from 1.93 to 1.46 (Figure 6b) with a decrease ratio of 24.4%. Due to the amount of light scattering that TiO_2_ used in the blue bottle solution, the absorbance value in Figure 6 was high (greater than one) [20].

The photo-generated electrons reduced MB (blue) to colorless LMB during photocatalysis, and the degree of photocatalytic reaction was reflected in the absorbance of MB. The reduced absorption value for sample T4 was greater than that of sample T2 with the same UV intensity and the same irradiation time, indicating sample T4 had better photocatalytic performance.

### 3.3. Characterization of Printability

According to the above analysis, sample T4 had the best photocatalytic properties. T4 responded to UV irradiation and reduced MB quickly, which would help improve oxygen indicator performance. Thus, T4 was selected to prepare the oxygen indicator. In order to use the indicator via screen printing, we studied the printability of the oxygen indicator solution including rheological properties, thixotropic properties, and wettability.

**Figure 7:**
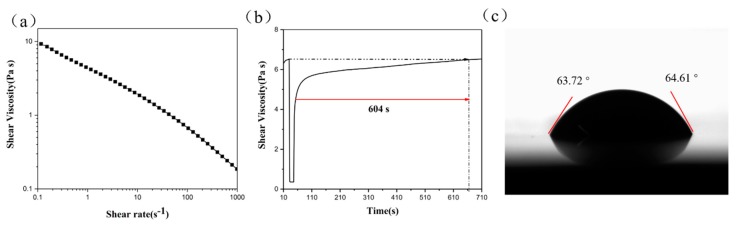
(**a**) Rheological curve, (**b**) thixotropy curve, and (**c**) contact angle of the oxygen indicator solution.

The rheological properties of the oxygen indicator were investigated by measuring the shear viscosity as a function of shear rate, as presented in Figure 7a. The shear viscosity decreased quickly with increasing shear rate because the increasing shear rate impaired the adhesion between particles and destroyed the ink structure, thereby diluting the viscosity. The results indicated that the oxygen indicator solution was a pseudo-plastic fluid. As for screen-printing, the phenomenon of shear thinning is beneficial to the smooth transfer of ink [23,24].

During the printing process, the shear rate of the solution was changeable, due to the damage and recovery of the solution structure. Figure 7b shows the thixotropy pattern of the oxygen indicator solution. When the shear rate changed from 0.1 s^−1^ to 1 000 s^−1^, the viscosity decreased quickly. This phenomenon of shear thinning was consistent with the results of the rheological analysis. When the shear rate changed from 1 000 s^−1^ to 0.1 s^−1^, the damaged structure gradually recovered and the recovery time of the viscosity approached 604 s. Some recovery time contributed improving the clarity of the printed image.

The contact angle is a measurement of the wettability of a solution to a substrate’s surface. The smaller the contact angle, the better the wettability to the substrate [25]. As shown in Figure 7c, the left and right contact angles were 63.72° and 64.61°, respectively, both of which were less than 90°, indicating that the oxygen indicator ink possessed satisfying wettability on the PET film.

### 3.4. Application of Oxygen Indicator in MAP

**Figure 8:**
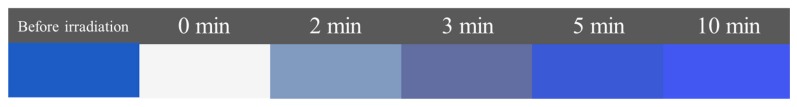
The color change in the oxygen indicator during recovery (in air).

After printing on the PET film, we investigated the color change in the oxygen indicator during the recovery stage in the air, as shown in Figure 8. The initial color was blue, and after UV irradiation (as 0 min) it turned white. Then, two minutes later, the indicator was light blue. As time went on, the color deepened. The color changed back to its original blue after about five minutes. The results showed that the prepared oxygen indicator effectively responded to the oxygen.

**Figure 9:**
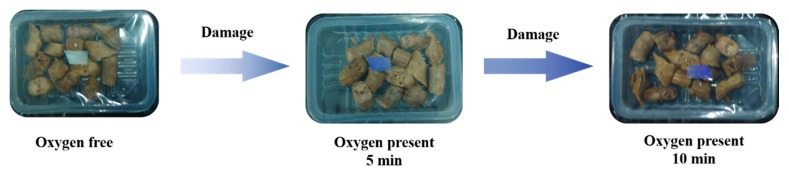
Color change of oxygen indicator in packaging of duck neck (10 min, room temperature).

Then, the printed PET film (as an oxygen indicator label) was sealed in duck neck MAP, full with 100% N_2_. The packaging was irradiated under UV light for one minute. The color changes of the label under breakage conditions (10 min, room temperature) are presented in Figure 9. When the package was damaged, the oxygen concentration gradually increased inside the packaging. The label’s color turned from white to light blue in five minutes. After 10 min, the color turned deep blue. The results indicated that the oxygen indicator label had different color responses to different oxygen concentrations, and quickly indicate the integrity of the packaging.

Hence, the prepared oxygen indicator label performed well as a colorimetric indicator for monitoring the integrity of MAP. Merchants can estimate the quality of the package content according to the color of the label, and adjust the sales strategy accordingly. For consumers, the quality of the products can be easily judged by color changes, effectively avoiding food safety problems caused by broken packaging. Simply, the oxygen indicator label can provide risk warnings for both consumers and businesses alike, as has been demonstrated previously using P25 TiO_2_ [7].

## 4. Conclusions

We reported the production of a colorimetric oxygen indicator based on TiO_2_ nanotubes incorporated with glycerol, methylene blue, and HEC. The indicator enables the visual judgement of intact packaging through color changes, where blue indicates leaking packaging. The indicator was easy to print, applicable for inside packaging by screen printing. Given the satisfactory properties of the TiO_2_ material, large specific surface (190.35 m^2^/g) area, and wide band gap (3.34 eV), the prepared indicator responded quickly during activation stage and was not affected by natural light. Thus, the efficiency and accuracy of the indicator were proven.

## Figures and Tables

**Table 1:** Synthesis conditions of TiO_2_ nanotube samples during microwave-assisted hydrothermal.

Sample	Temperature (°C)	Time (h)	Calcination
T1	130	2	No
T2	130	2	Yes
T3	130	3	No
T4	130	3	Yes
